# Reported COVID-19 vaccines side effects among Jordanian population: a cross sectional study

**DOI:** 10.1080/21645515.2021.1981086

**Published:** 2021-10-06

**Authors:** Haya Omeish, Angam Najadat, Sayer Al-Azzam, Nada Tarabin, Amer Abu Hameed, Neebal Al-Gallab, Hadeel Abbas, Lana Rababah, Majd Rabadi, Reema Karasneh, Mamoon A. Aldeyab

**Affiliations:** a Department of Internal Medicine, Royal Jordanian Medical Centre- Amman-Jordan; b Department of Clinical Pharmacy, Faculty of Pharmacy, Jordan University of Science and Technology, Irbid, Jordan; c Department of Medicine, Jordan University Hospital, Amman, Jordan; d Faculty of Medicine, Yarmouk University, Irbid, Jordan; e Faculty of Medicine, University of Jordan, Amman, Jordan; f Department of Dentistry, Ministry of Health, Amman, Jordan; g Department of Basic Medical Sciences, Faculty of Medicine, Yarmouk University, Irbid, Jordan; h Department of Pharmacy, School of Applied Sciences, University of Huddersfield, Huddersfield, UK

**Keywords:** COVID-19 vaccine, side effects, COVID-19, symptoms, vaccine

## Abstract

Concerns about the safety and side effects of coronavirus SARS CoV2 vaccines have been raised among many communities worldwide. The aim of this study was to describe the side effects reported by vaccinated individuals in Jordan. A cross-sectional survey was used to recruit responses from participants who were vaccinated with either one dose or both doses of any of the administered vaccines in Jordan (AstraZeneca, Pfizer, Sinopharm). A total of 1,086 participants were enrolled in the study. Most of participants have not been infected with SARS CoV2 before receiving the vaccine (77.2%). Larger proportion of the study population received Pfizer vaccine (40.6%) followed by the AstraZeneca vaccine (33.0%), and Sinopharm vaccine (26.4%). Side effects after receiving the first dose of the vaccine were reported by most participants (89.9%) and included pain at the injection site (78.4%), fatigue (51.8%), myalgia (37.6%), headache (33.1%), and chills (32.3%). To a lesser extent, there were gastrointestinal side effects such as nausea (15.1%), loss of appetite (9.4%), and diarrhea (6.4%). More side effects were significantly associated with AstraZeneca vaccine (*P* < .001). Only one case for each of second dose of Pfizer and Sinopharm vaccines reported that their side effects required hospitalization. In this study, we found that people in Jordan experienced more side effects with AstraZeneca vaccine followed by Pfizer vaccine and the least one is Sinopharm vaccine. Our study showed that these side effects are not severe and should not be an obstacle against the successful control of the pandemic in Jordan.

## Introduction

Severe acute respiratory syndrome coronavirus 2 (SARS-CoV-2) is the cause of coronavirus disease 2019 (COVID-19). Since its emergence in December 2019, it caused a global humanitarian crisis; affecting health, economy, and education in addition to other challenges.^1^

In the wake of the pandemic, most of the countries including Jordan have imposed protective measures such as wearing masks, distancing, stay-at-home-strategy, and lockdown, but these measures are not going to persist. Therefore, in order to manage, reduce, and eradicate COVID-19 infection, therapeutic and preventative solutions are required. Regarding pharmacologic therapy for SARS-CoV-2, studies on the use of preexisting medications (e.g., hydroxychloroquine and remdesivir) for the treatment of COVID-19 were contradictory and did not confirm a conclusion.^2,3^ This highlighted the need for specific antivirals against SARS-COV-2 to be developed and authorized to control the pandemic.^4^ In terms of preventative measures, population immunity is needed to be achieved once the disease has spread to become a pandemic.^5^ However, waiting for natural population immunity through infection with the virus to achieve herd immunity is not ethical or acceptable. Therefore, extended vaccination campaigns were found to be the only accepted way for developing population immunity.^6,7^

By the relentless work of governments and scientists, and depending on advanced biotechnology and interim analyses, COVID-19 vaccines have been developed within a year of the first reports of COVID-19.^8^ In December 2020, the U.S. Food and Drug Administration authorized the first emergency use authorization (EUA) for Pfizer-BioNTech COVID-19 Vaccine.^9^ Afterward, Jordanian government initiated a vaccination campaign on January 13, 2021 and by April 25, 2021, 807,175 vaccine doses have been administered in Jordan.^10^ The emergency use of five types of vaccines was approved by the Jordanian Food & Drug Administration. The approved vaccines were Pfizer-BioNTech’s BNT162b2, AstraZeneca’s AZD-1222, Sinopharm’s BBIBP-CorV, Johnson & Johnson’s Ad26.COV2.S, and Sputnik vaccines.^11^ AstraZeneca, Johnson & Johnson, and Sputnik V are vaccines using adenoviral vectors with the efficacy of 81%, 66%, and 92%, respectively.^12,13^ Pfizer-BioNTech is a nucleic acid-based mRNA vaccine with an efficacy of 95%.^14^ Sinopharm is an inactivated vaccine with an efficacy of 79%.^15^

No vaccine is totally free from any side effects or complications.^16^ Early side effects either local ones like pain, redness, and swelling, or the systemic ones like headache, nausea, tiredness, myalgia, chills, fever are expected with any vaccines.^17^ However, there may be other serious side effects such as anaphylaxis to a vaccine component, which was reported to be secondary to allergic reactions to polyethylene glycol (PEG).^18^ In addition, blood clotting events were suggested to be caused secondary to the administration COVID-19 vaccines including AstraZeneca, Pfizer, and Moderna vaccines.^19,20^

Side effects of vaccines may be reported through the government’s reporting systems such as the Vaccine Adverse Event Reporting System (VAERS) in the US and Yellow card in the UK.^21,22^ In Jordan, people who received the vaccine are encouraged to report any observed side effects to the Ministry of Health’s platform through the link included in the SMS received upon registration.^23^ However, the purpose of reporting is to assure the safety of the vaccinated person rather than providing an early warning about the safety of the vaccine or if it may require further investigation.^23^ Therefore, in this study we aimed to identify vaccine side effects reported among Jordanian population who have received one of the earliest administered vaccines (Pfizer, AstraZeneca, and Sinopharm) in Jordan.

## Patients and methods

### Study design

A cross-sectional (online survey) study was conducted including subjects who have been vaccinated with either the first dose or both doses of any of the administered vaccines in Jordan (Pfizer, AstraZeneca, and Sinopharm). Data collection was carried out between March 13 and April 23, 2021.

### Study instrument

The questionnaire was created after extensive review of the literature using Google Forms and later distributed through social media platforms. Participants were asked whether they have received COVID-19 vaccines (Pfizer, AstraZeneca, or Sinopharm) and if they answered “yes,” they will be able to complete the questionnaire. “Required” and “Limit to one response” authentication options were applied to minimize missing data and to avoid duplicate responses. Face and content validity of the survey were examined by five experts in the field. Participants were given a brief description of the study and were informed that all responses were voluntary, anonymized, and treated as confidential. The study protocol was approved by the institutional review board at Yarmouk University (IRB/2021/23).

The survey included two parts; The first part was collecting demographic information of participants, including marital status, age, gender, education level, health status, and history of COVID-19 infection. Participants were not asked whether that COVID-19 infection was confirmed by PCR or not. However, self-reporting of COVID-19-related symptoms was found to have high epidemiological evaluation ability and was comparable to confirmed PCR findings.^24,25^ The second part was collecting information about self-reported side effects of COVID-19 vaccines. The survey asked the participants what type of COVID-19 vaccine they had received and whether they had received one or two doses of the vaccine.

### Statistical analysis

Data collected in Google Forms was exported to Microsoft Excel file, which was directly imported into IBMSPSS® 24.0 for statistical analysis. Descriptive statistics were used to describe the socio-demographic characteristics of participants. Associations between categorical variables were evaluated by performing Pearson chi-square test. The statistical significance level was set at *P* < .05. Cronbach’s alpha coefficient was applied to test the internal consistency of the survey items. According to Ministry of Health of Jordan statistics, by March 25, 2021, 273,900 individuals had received the first dose of COVID-19 vaccines and 75,921 individuals had received the second dose. Sample size was calculated for this study with 5% margin of error and a 95% confidence level via web-based sample size calculator (Raosoft®). The minimum recommended sample size was 384.

## Results

### Population characteristics

Sample characteristics of 1,086 individuals who were enrolled in the study are presented in Table 1. Results showed that most participants were females (63.0%) and had diploma degree (81.8%). Two thirds of participants (78.8%) reported having no chronic diseases. A minority of the participants (18.9%) were previously infected with the SARS-CoV-2 virus and only (24.3%) of participants reported having received annual influenza vaccine. With regard to the type of COVID-19 vaccination, more than one third of the study participants (40.6%) received Pfizer vaccine, while 33.0% received AstraZeneca vaccine, 26.4% received Sinopharm vaccine, and the majority of participants (72.5%) got the first dose only. Those who reported the presence of side effects after receiving their first dose of the vaccine were 89.9% and 75.9% after the second dose. However, none of participants reported that they required hospitalization after the first dose, and only 0.7% after the second dose of any of COVID-19 vaccines. Regarding medications used by participants for symptom relief after vaccination, paracetamol (500–1000 mg) was mostly used (41.8%) after receiving the first dose. Azithromycin was also used by 10.8% of participants after the first dose of receiving COVID-19 vaccination.

**Table 1.** Study population characteristics (N = 1086)

Variable		N (%)		P value
**Gender**	Female Male	684 (63.0) 402 (37.0)		<0.001
**Age**	18–24 years 25–65 years >66 years	180 (16.6) 869 (80.0) 37 (3.4)		<0.001
**Marital status**	Single Married	539 (49.6) 547 (50.4)		<0.001
**Education level**	School Diploma University Other	74 (6.8) 888 (81.8) 83 (7.6) 41 (3.8)		<0.001
**Presence of chronic disease**	No Yes	856 (78.8) 230 (21.2)		<0.001
**Allergy**	No allergy Seasonal allergy Food allergy Penicillin allergy	805 (74.1) 232 (21.4) 23 (2.1) 25 (2.3)		<0.001
**Receive annual influenza vaccine**	No Yes	813 (74.9) 264 (24.3)		<0.001
**COVID19 vaccine**	First dose only First and second dose	787 (72.5) 299 (27.5)		<0.001
**Type of COVID19 vaccine**	Pfizer Sinopharm AstraZeneca	441 (40.6) 287 (26.4) 358 (33.0)		<0.001
**History of COVID19 infection**	No Yes, before the vaccine. Yes, after the 1^st^ dose Yes, after the 2^nd^ dose	838 (77.2) 205 (18.9) 38 (3.5) 5 (0.5)		<0.001
		**1^st^ dose**	**2^nd^ dose**	**P value**
**Having any side effect**	No Yes	110 (10.1) 976 (89.9)	72 (24.0) 227 (75.9)	0.101
**Require hospitalization**	No Yes	1041(95.9) 0 (0.0)	297 (99.3) 2 (0.7)	0.310
**Onset of symptoms**	Immediately 1^st^ day 1^st^ week 2^nd^ week 3^rd^ week	95 (8.7) 781(71.9) 68 (6.3) 10 (0.9) 4 (0.4)	27 (9.7) 190 (63.5) 21 (7.0) 38 (12.7) 1 (0.3)	0.358
**Medications used for symptoms relief after vaccination**	Paracetamol (500–1000 mg) NSAIDS(ibuprofen, Diclofenac) Antihistamine Multivitamins Aspirin Esomeprazole Azithromycin Not receive any medication	454(41.8) 26 (2.4) 3 (0.3) 4 (0.4) 9 (0.8) 2 (0.2) 3 (0.3) 117 (10.8)	90 (8.3) 4 (0.4) 2 (0.2) 0 (0.0) 1 (0.1) 0 (0.0) 3 (0.3) 28 (2.6)	0.251

NSAIDS: Non-steroidal anti- inflammatory drugs;N: population size.

### Side effects after receiving Covid-19 vaccines related to the dose

As shown in Table 2, most participants (78.4%) suffered from pain in addition to swelling and redness at the injection site (16.3%). More than one third of the participants (37.6%) suffered from bone and muscles pain after the first dose. In comparison, lower proportions were observed after receiving the second dose.

**Table 2.** Side effect after receiving vaccines related to the dose

	First dose N (%) N = 1086	Second dose N(%) N = 299	P value
**Local symptoms** Injection site pain Swelling and redness of injection site	851 (78.4) 177 (16.3)	200 (66.8) 45 (15.0)	0.004 0.216
**Bone and muscle pain**	408 (37.6)	85 (28.4)	<0.001
**Flu like symptoms** Fever Headache Chills Sore throat Fatigue Cough Runny nose Loss of taste Loss of smell	300 (27.6) 359 (33.1) 351 (32.3) 90 (8.3) 563 (51.8) 91 (8.4) 76 (7.0) 14 (1.3) 16 (1.5)	57 (19.1) 59 (19.7) 69 (23.1) 18 (6.0) 121 (40.4) 10 (3.3) 14 (4.6) 2 (0.7) 3 (1.0)	<0.001 <0.001 <0.001 0.031 <0.001 0.048 0.115 0.264 0.428
**GI side effect** Nausea Diarrhea Vomiting Loss of appetite Abdominal pain Constipation	164 (15.1) 70 (6.4) 30 (2.8) 102 (9.4) 65 (6.0) 7 (0.6)	23 (7.7) 13 (4.3) 8(2.7) 19 (6.3) 7 (2.3) 2 (0.7)	<0.001 0.022 0.003 <0.001 <0.001 0.431
**Psychological side effect** Sleep disturbance Anxiety and stress Depression	110 (10.1) 68 (6.3) 48(4.4)	22 (6.7) 8 (2.4) 6 (1.8)	<0.001 0.003 0.007
**Irregular menses (Females)**	17 (1.6)	4 (1.3)	0.861
**Decreased libido**	14 (1.3)	5 (1.6)	0.264
**Lymph node enlargement and swelling**	12 (1.1)	4 (1.3)	0.044
**Eye symptoms** Blurred vision	40 (3.7)	8 (2.7)	0.011
**Skin itching** **Acne** **Sweating**	29 (2.7) 22 (2.0) 76 (7.0)	6 (2.0) 5 (1.6) 11 (3.7)	0.209 0.978 0.001
**Increase urination** **Change urine color**	16 (1.5) 11 (1.0)	2 (0.7) 2 (0.7)	0.428 0.985
**Cardiac side effect** Shortness of breath Palpitation Chest pain Loss of consciousness Paleness Bleeding anywhere	76 (7.0) 93 (8.6) 56 (5.2) 7 (0.6) 35 (3.2) 5 (0.5)	21 (7.0) 10 (3.3) 11 (3.7) 0 (0.0) 6 (2.0) 0 (0.0)	0.017 0.010 0.023 0.362 0.311 0.167
**Dizziness**	157 (14.5)	27 (9.0)	<0.001

In general, fever was reported by 27.6% individuals after receiving the first dose of vaccine, and 19.1% after the second dose. Almost half of participants (51.8%) reported fatigue after the first dose, and 40.4% after the second dose. Headache was also reported by one third (33.1%) of individuals after the first dose of vaccine and by 19.7% after the second dose. In addition, higher proportions of participants suffered from chills after receiving the first dose (32.3%) compared to second dose (23.1%) The most reported gastrointestinal side effect was nausea (15.1%) after receiving the first dose of vaccination, and only (7.7%) was reported after receiving the second dose. Vomiting, diarrhea, and constipation were less reported after first and second dose of vaccination. In general, higher proportions of participants reported vaccine side effects after receiving the first dose compared to those reported after the second dose.

### Reported COVID-19 vaccine side effects and their correlation with participant’s gender

Results in Table 3 show a significant difference (*P* = .001) between female participants, who suffered from COVID-19 vaccine side effects after the first dose of vaccine (92.3%), and male participants (85.8%). However, no significant difference was shown after receiving the second dose. Specifically, there was a significant increase (*P* < .001) in the number of females (83%) who reported pain at the injection site after receiving the first dose of vaccines in comparison to males (70.4%). Nonetheless, no significant difference was shown with the second dose. Headache was significantly higher among females after receiving the first dose (*P* = .003) and the second dose (*P* = .030). Regarding cardiac side effects, palpitation was significantly higher among females than it for males (*P* < .05).

**Table 3.** Side effect after receiving vaccines related to gender

**Symptoms**	**After 1^st^ dose of COVID-19 vaccine**Frequency (%)		p Value	**After 2^nd^ dose of COVID-19 vaccine**Frequency (%)		p Value
	Female N = 684	Male N = 402		Female N = 211	Male N = 109	
**Presence** No Yes	53 (7.7) 631 (92.3)	57 (14.2) 345(85.8)	0.001	54(25.6)15 7 (74.4)	18(16.5) 91 (83.5)	0.065
**Injection site pain** **Redness and swelling**	568 (83.0) 129 (18.9)	283 (70.4) 48 (11.9)	<0.001 0.003	125 (59.2) 33 (15.6)	75 (68.8) 12 (11.0)	0.872 0.142
**Bone and muscle pain**	264 (38.6)	144 (35.8)	0.362	57 (35.5)	28 (25.6)	0.418
**Flu like symptoms** Fever Headache Chills Sore throat fatigue cough runny nose loss of taste loss of smell	174(25.4) 248 (36.3) 244 (35.7) 73 (10.7) 381 (55.8) 70 (10.2) 58 (8.5) 12 (1.8) 14 (2.0)	126 (31.3) 111 (27.6) 107 (26.6) 17 (4.2) 182 (45.3) 21 (5.2) 18 (4.5) 2 (0.5) 2 (0.5)	0.036 0.003 0.002 <0.001 0.004 0.013 0.076 0.041	38(18.0) 45(21.3) 47(22.2) 15(7.1) 85(40.2) 7(3.3) 8 (3.7) 2 (0.9) 3 (1.4)	19 (17.4) 14(12.8) 22(20.1) 3 (2.7) 36 (33.0) 3(2.7) 6 (5.5) 0 (0.0) 0 (0.0)	0.554 0.030 0.362 0.071 0.079 0.644 0.649 0.278 0.184
**GI side effect** Nausea Diarrhea Vomiting loss of appetite abdominal pain constipation	127(18.6) 52(7.6) 18(2.6) 74 (11.1) 53 (7.7) 6 (0.9)	37 (9.2) 18(4.5) 12(3.0) 28 (7.2) 12 (3.0) 1 (0.2)	<0.001 0.043 0.731 0.039 0.001 0.211	16 (7.5) 9(4.2) 7 (3.3) 14(6.6) 4 (1.8) 1 (0.4)	7 (6.4) 4(3.6) 1 (0.9) 5(4.5) 3 (2.7) 1 (0.9)	0.509 0.639 0.149 0.330 0.748 0.703
**Psychological SE** Sleep disturbance Anxiety and stress Depression	74 (10.8) 47 (6.9) 35 (5.1)	36 (9.0) 21 (5.2) 13 (3.2)	0.326 0.279 0.145	17(8.0) 6(2.8) 4(1.8)	5(4.5) 2(1.8) 1(0.9)	0.161 0.480 0.430
**Irregular menses**	17 (2.5)	0 (0.0)	0.001	4 (1.8)	0 (0.0)	0.125
**Decreased libido**	4 (0.6)	10 (2.5)	0.007	4(1.8)	1(0.9)	0.430
**Lymph node enlargement & swelling**	8 (1.2)	4 (1.0)	0.790	2(0.9)	2 (1.8)	0.590
**Skin itching** **Acne** **Sweating**	26(3.8) 20 (2.9) 45 (6.6)	3 (0.7) 2 (0.5) 31 (7.7)	0.003 0.006 0.480	5 (2.3) 5 (0.7) 9 (4.2)	1 (0.9) 0 (0.0) 2 (1.8)	0.301 0.086 0.193
**Increase urination** **Change urine color**	9 (1.3) 6 (0.9)	7 (1.7) 5 (1.2)	0.574 0.560	1 (0.1) 2(0.3)	1 (0.2) 0 (0.0)	0.703 0.278
**Blurred vision**	30 (4.4)	10 (2.5)	0.109	8 (1.2)	0 (0.0)	0.030
**Cardiac side effects** Shortness of breath Palpitation Chest pain Loss of consciousness **Paleness** **Bleeding anywhere**	56 (8.2) 72 (10.5) 39 (5.7) 4 (0.6) 24 (3.5) 4 (0.6)	20 (5) 21 (5.2) 17 (4.2) 3 (0.7) 11 (2.7) 1 (0.2)	0.045 0.003 0.289 0.748 0.486 0.430	14 (6.6) 6 (2.8) 8 (3.7) 0 (0.0) 5 (0.7) 0 (0.0)	7 (6.4) 4(3.6) 3(2.7) 0 (0.0) 1 (0.2) 0 (0.0)	0.724 0.844 0.501 – 0.301 –
**Dizziness**	119 (17.4)	38 (9.5)	<0.001	20 (9.4)	7 (6.4)	0.227
**Require hospitalization**	0 (0.0)	0 (0.0)	0.260	2 (1.0)	0 (0.0)	0.241
**Onset of Symptoms** Immediately 1^st^ day 1^st^ week 2^nd^ week 3^rd^ week	69(11.0) 506(80.7) 44 (7.0) 6(1.0) 2(0.3)	26 (7.9) 275 (83.1) 24 (7.3) 4 (1.2) 2 (0.6)	0.577	20(11.4) 120(68.2) 16(9.1) 19(10.8) 0 (0.0)	7(6.9) 70(68.6) 5(4.9) 19(18.6) 1(1)	0.153
**Duration of Symptoms** **mean (SD)**	2.32(2.3)	2.64 (4.5)	0.082	2.82 (3.74)	2.54 (4.1)	0.354

N: population size, SD: standard deviation.

### Side effect after receiving vaccines related to vaccine type

Table 4 shows reported side effect after receiving vaccines related to vaccine type. It has been shown that the first dose of AstraZeneca vaccine was significantly associated with higher reports of bone and muscle pain, flu-like symptoms, gastrointestinal (GI) symptoms, psychological symptoms, cardiac symptoms, and dizziness compared to Sinopharm and Pfizer vaccines. Nevertheless, earlier onset of symptoms was observed after receiving the first dose of Pfizer in comparison to AstraZeneca and Sinopharm vaccines (Table 5).

**Table 4.** Side effect after receiving vaccines related to vaccine type

Symptoms	First dose of COVID19 vaccine				Second dose of COVID19 vaccine			
	Pfizer N = 441	AstraZeneca N = 358	Sinopharm N = 287	P value	Pfizer N = 175	AstraZeneca N = 0	Sinopharm N = 124	P value
Bone and muscle pain	116 (26.3)	237 (66.2)	55 (19.2)	<0.001	73 (41.7)	0(0.0)	12 (9.6)	<0.001
Local								
Injection site pain	385 (87.3)	300 (83.8)	166 (57.8)	<0.001	148 (84.5)	0 (0.0)	52 (41.9)	<0.001
Local swelling and redness	81 (18.4)	84 (23.5)	12 (4.2)	<0.001	41 (23.4)	0 (0.0)	4 (3.2)	<0.001
Flu like								
Fever	65 (14.7)	203 (56.7)	32 (11.1)	<0.001	52 (29.7)	0 (0.0)	5 (4.0)	<0.001
Chills	93 (21.1)	220 (61.5)	38 (13.2)	<0.001	64 (36.5)	0 (0.0)	5 (4.0)	<0.001
Fatigue	197 (44.7)	274 (76.5)	92 (32.2)	<0.001	96 (54.8)	0 (0.0)	25 (20.1)	<0.001
Headache	113 (25.6)	184 (51.4)	62 (21.6)	<0.001	49 (28.0)	0 (0.0)	10 (8.0)	<0.001
Cough	27(6.1)	39(10.9)	25(8.7)	0.052	7 (4.0)	0 (0.0)	3(2.4)	0.141
Sore throat	31(7.0)	36 (10.1)	23(8.0)	0.298	15 (8.5)	0 (0.0)	3 (2.4)	0.001
Runny nose	24 (5.4)	39 (10.9)	13 (4.5)	0.002	10 (5.7)	0 (0.0)	4 (3.2)	0.018
Loss of taste	3 (0.7)	8 (2.2)	3 (1.0)	0.140	2 (1.1)	0(0.0)	0 (0.0)	0.231
Loss of smell	3 (0.7)	9 (2.5)	4 (1.4)	0.101	3 (1.7)	0 (0.0)	0 (0.0)	0.111
GI								
Loss of appetite	23 (5.4)	63 (17.8)	16 (5.7)	<0.001	17 (9.7)	0 (0.0)	2 (1.6)	<0.001
Nausea	42 (9.5)	100 (27.9)	22 (7.7)	<0.001	21 (12.0)	0 (0.0)	2 (1.6)	<0.001
Vomiting	4 (0.9)	24 (6.7)	2 (0.7)	<0.001	7 (4.0)	0 (0.0)	1 (0.8)	0.023
Abdominal pain	16(3.6)	38(10.6)	11(3.8)	<0.001	5 (2.8)	0 (0.0)	2 (1.6)	0.248
Diarrhea	19(4.3)	38(10.6)	13(4.5)	<0.001	10 (5.7)	0 (0.0)	3 (2.4)	0.024
Constipation	3(0.7)	3(0.8)	1(0.3)	0.737	0 (0.0)	0 (0.0)	2 (1.6)	0.494
Psychological								
Anxiety	18 (4.1)	35 (9.8)	15 (5.2)	0.003	6 (3.4)	0 (0.0)	2 (1.6)	0.138
Depression	14 (3.2)	23 (6.4)	11 (3.8)	0.072	3 (1.7)	0 (0.0)	2(1.6)	0.393
Sleep disturbance	23 (5.2)	70 (19.6)	17 (5.9)	<0.001	18 (10.2)	0 (0.0)	4(3.2)	<0.001
Irregular menses	7 (1.6)	8 (2.2)	2 (0.7)	0.294	2 (1.1)	0 (0.0)	2 (1.6)	0.559
Cardiac								
Loss of consciousness	3(0.7)	3(0.8)	1(0.3)	0.737	0 (0.0)	0 (0.0)	0 (0.0)	-
Palpitation	24(5.4)	53(14.8)	16(5.6)	<0.001	8 (4.5)	0 (0.0)	2 (1.6)	0.025
Chest pain	15(3.4)	31(8.7)	10(3.5)	0.001	9 (5.1)	0 (0.0)	2(1.6)	0.020
Shortness of breath	19(4.3)	41(11.5)	16(5.6)	<0.001	15 (8.5)	0 (0.0)	6 (4.8)	0.006
Paleness	11 (2.5)	19 (5.3)	5(1.7)	0.021	5 (2.8)	0 (0.0)	1 (0.8)	0.085
Bleeding anywhere	1 (0.2)	3 (0.8)	1 (0.3)	0.424	0 (0.0)	0 (0.0)	0 (0.0)	-
Dizziness	37 (8.4)	88 (24.6)	32 (11.1)	<0.001	23 (13.1)	0 (0.0)	4 (3.2)	<0.001
Decreased libido	5(1.1)	7 (2.0)	2(0.7)	0.346	3 (1.7)	0 (0.0)	2 (1.6)	0.291
Lymph node enlargement	5 (1.1)	6 (1.7)	1 (0.3)	0.276	4 (2.2)	0 (0.0)	0 (0.0)	0.053
Eye (blurred vision)	8 (1.8)	22 (6.1)	10 (3.5)	0.005	6 (3.4)	0 (0.0)	2 (1.6)	0.082
Skin symptoms								
Itching	10 (2.3)	14 (3.9)	5 (1.7)	0.188	4 (2.2)	0 (0.0)	2 (1.6)	0.212
Acne	10 (2.3)	8 (2.2)	4 (1.4)	0.675	4 (2.2)	0 (0.0)	1 (0.8)	0.161
Sweating	14 (3.2)	57 (15.9)	5 (1.7)	<0.001	9 (5.1)	0 (0.0)	2 (1.6)	0.014
Increase urination	5 (1.1)	11 (3.1)	0 (0.0)	0.004	2 (1.1)	0 (0.0)	0 (0.0)	0.231
Change urine color	4 (0.9)	5 (1.4)	2 (0.7)	0.650	2 (1.1)	0 (0.0)	0 (0.0)	0.231

**Table 5.** Side effects of the three types of COVID19 vaccine

Variable	First dose of COVID19 vaccine				Second dose of COVID19 vaccine		
	Pfizer N = 441	AstraZeneca N = 358	Sinopharm N = 287	P value	Pfizer N = 175	Sinopharm N = 124	P value
Presence of symptoms	409(92.7)	330 (92.2)	237 (82.6)	<0.001	159(90.8)	57 (45.9)	0.034
Require hospitalization	0(0.0)	0(0.0)	0 (0.0)	-	1 (0.5)	1 (0.8)	0.085
Onset of symptoms				0.012			<0.001
Immediately	46 (10.4)	20(5.6)	29 (10.1)		16 (9.1)	9 (7.2)	
1st day	321(72.8)	301(84.1)	159 (55.4)		134 (76.5)	53 (42.7)	
1^st^ week	31(7.0)	17(4.7)	20 (7.0)		12 (6.8)	9 (7.2)	
2^nd^ week	5(1.1)	2(0.6)	3 (1.0)		10 (5.7)	20 (16.4)	
3^rd^ week	1(0.2)	1(0.3)	2 (0.7)		1 (0.5)	0 (0.0)	
>30 days	0(0.0)	0(0.0)	0(0.0)		0(0.0)	0 (0.0)	
Duration of symptoms (days)Mean (SD)	2.59(3.6)	1.67(0.57)	2.02(1.67)	0.31	2.56(3.6)	3.32(4.6)	0.33
Min-Max	0–30	0–30	0–10		0–30	0–21	

SD: standard deviation.

A logistic regression was performed to ascertain the effects of age, marital status, gender, education, allergy, health condition, receiving influenza vaccine, previous infection of COVID-19 and the type of vaccine on the likelihood that participants have side effects after getting the vaccine. Female gender, increasing age, and getting the first dose were associated with an increased likelihood of having side effects after vaccination, *p* < .05.

Side effect after receiving vaccines related to age, influenza vaccine, health condition, allergy status, and history of COVID-19 infection are presented in Tables S1-S5 (supplementary material).

## Discussion

Our study showed that several side effects have been reported after receiving COVID-19 vaccination, mainly including pain at the injection site, flu-like symptoms and GI symptoms, and particularly after receiving the first dose of vaccination. The reported side effects were significantly higher among female participants and those who received AstraZeneca vaccination. Gender differences were also observed in Menni*et al*. (2021) study that included Pfizer and AstraZeneca vaccines recipients.^26^ This was also emphasized in a two phased randomized clinical trial that was conducted in China on Sinopharm vaccine, exhibiting more common side effects in females (55%) compared to males (45%).^27^ On the other hand, more severe symptoms associated with AstraZeneca vaccine administration were reported in a study that included 1,440 and 80 healthcare workers who received one dose of Pfizer and AstraZeneca COVID-19 vaccines, respectively.^28^

By comparing reported symptoms after receiving the first dose of vaccine with those reported after receiving the second dose, several studies on Pfizer and AstraZeneca vaccines showed that the prevalence of local and systemic side effects were higher after receiving the second dose compared to the first dose.^14,17,29–32^ On the contrary, our data are not consistent with this trend, as side effects were more frequent after receiving the first dose rather than the second one except for chills, sexual disturbance, and lymph node enlargement. However, this may be attributed to lower proportions in our population who have received the second dose particularly for those who have received AstraZeneca vaccine.

Similar to our study, local injection site symptom was the most common side effect with the same pattern observed in the clinical trial on BNT162b2 mRNA Covid-19 vaccine.^14^ This was also observed in a randomized, cross-sectional study in which 88.04% of participants reported local pain compared to other local site side effects.^33^ Moreover, a study on healthcare workers in the Czech Republic showed that injection site pain was the most common side effect experienced by participants.^29^ These findings were also consistent with the FDA’s report on Pfizer vaccine side effects and that reported in Ramasamy *et al*. (2020) study with the administration of AstraZeneca vaccine.^17,31^ Although with lower proportions than those observed with Pfizer or AstraZeneca vaccines administration, local symptoms were also the most common reported symptom with the administration of Sinopharm vaccine. This is consistent with the findings of Henan Province randomized controlled clinical trial that included 320 healthy participants who aged 18–59 years, where injection site pain was the complaint of 14 of 96 participant (14.5%) and 21 of 224 (9.37%) in phase 1 and phase 2, respectively.^34^

Consistent with our findings, fatigue was reported in several studies with the administration with Pfizer or AstraZeneca vaccines.^14,17,29,31–33^ However, higher proportions of those who received Sinopharm vaccine in our study complained from fatigue compared to that observed in Xia et al. (2021) randomized controlled trial.^27^ This is also consistent with the findings of Wu et al (2021) recent review of 87 publications with safety data from clinical trials and post-authorization studies of 19 COVID-19 vaccines.^35^

Gastrointestinal symptoms, particularly nausea, were reported in 23.% of those who received AstraZeneca vaccine with lower proportions observed with the administration of Pfizer vaccine in two cross sectional studies (15.94% and 13.0%) and one randomized trial with the administration of Sinopharm vaccine (1%).^27–29,33^ This trend was also observed in our study, though with higher proportions observed for AstraZeneca and Sinopharm vaccines and lower proportions with Pfizer vaccine. Nausea incidence in our study was also consistent with that observed in the WHO report with the administration of AstraZeneca vaccine.^32^

Other reported systemic reactions were fever and chills, which were higher in incidence in our study than that reported in a prospective observational study conducted in the UK with the administration of AstraZeneca vaccine.^26^ Furthermore, headache was observed among half of those who received either AstraZeneca or Pfizer vaccines.^28,29,33^ In fact, headache was the most prominent side effect of Pfizer vaccine in a real time analysis.^36^ However, these findings were consistent with ours for AstraZeneca but not Pfizer vaccines. These discrepancies may be attributed to population differences or methodologies used for reporting observed symptoms.

Fever, fatigue, headache, and myalgia were considered very rare side effects with the administration of Sinopharm vaccine in a randomized, phase 1 and phase 2 trials in China with reported fever (6%), fatigue (3%), headache (1%), and myalgia and joint pain (1%).^27^ In general, consistent with our findings, rates of local and systemic reactions were significantly lower among inactivated vaccines including Sinopharm vaccine compared to Pfizer and AstraZeneca vaccines as observed in Wu et al (2021) review.^35^

Notably, localized lymphadenopathy was reported in two cross-sectional studies that included healthcare workers who were given Pfizer vaccine.^29,33^ However, lower proportions were observed in our study perhaps due to lower health literacy among Jordanian population that might be affecting the ability to recognize lymphadenopathy symptoms compared to that expected from health care workers in the aforementioned studies.^37^

Dizziness was the only neurological symptom reported in our study. Other reported neurological manifestations were considered rare in Kadali *et al* (2021) study with the administration of Pfizer vaccine.^33^ Furthermore, higher proportions of psychological symptoms were observed in our population compared to that reported in Kadali *et al* (2021) study with less than 1% prevalence of stress and depression.^33^

Our study revealed that most of the symptoms were not severe to require hospitalization with the administration of either Pfizer or AstraZeneca vaccines and the duration of symptoms were only for few days after vaccination. The same trend was reported in several studies with most side effects were mild to moderate in severity and usually resolve within few days after vaccination.^14,31,32,36^

### Strengths and limitations

Our study utilized an online-based questionnaire that may introduce a source of selection bias. Despite, online web-based questionnaires were found to be cost effective method that represent total population and have the ability to reach people who are difficult to be reached.^38,39^ In fact, the availability of inexpensive wireless services in Jordan enhanced the use of smartphones that reached 90.3% of the population.^40,41^ This would facilitate the access to social media platforms that were progressively used particularly during the COVID-19 pandemic among Jordanian population as a source of information.^42^ Furthermore, age is less likely to affect our results particularly as social media, mainly Facebook, has also been found to enable elderly population to compensate for the lack of face-to-face contact during the pandemic.^43^ Moreover, this method of data collection, provides safe and private environment for the participants to answer precisely and truthfully in comparison with face-to-face interactions.^39^ Unfortunately, there is no published data that reflects the number of who received the vaccine by gender since the start of vaccination campaign in Jordan^44^ nor on the proportion of each vaccine used in the Jordanian population to assess the representativeness of our sample. However, this is less likely to affect our results particularly as the sample size is relatively large when compared to vaccinated population at time of data collection. Besides, the questionnaire was delivered in Arabic language to provide better interpretation. However, the survey was done very soon after starting vaccination in Jordan, therefore, responses regarding delayed symptoms may not have been reported.

## Conclusion

The most prevalent side effects for the three vaccines Pfizer, AstraZeneca, and Sinopharm were the local ones, including pain, redness and swelling at the injection site. Musculoskeletal pain, fever, chills, fatigue, headache, nausea and vomiting, and anxiety were documented more with AstraZeneca vaccine, followed by Pfizer vaccine, and then Sinopharm vaccine. Females were more prone to show symptoms than males after receiving COVID-19 vaccine. Overall, the reported symptoms were well-tolerated, however, further research and investigations of long-term symptoms and safety profiles are required.

## Supplementary Material

Supplemental Material
