# Supplementary material for: Reported COVID-19 vaccines side effects among Jordanian population: a cross sectional study
Source: Hum Vaccin Immunother. 2021 Oct 6;18(1):1981086. doi: 10.1080/21645515.2021.1981086 (PMC8920252; doi:10.1080/21645515.2021.1981086)
Supplement: Supplemental Material [file KHVI_A_1981086_SM5775.docx]

**Supplementary Table 1 - Side effect after receiving vaccines related to age**

| **Symptoms** | **After 1^st^ dose of COVID-19 vaccine**  Frequency (%) | | | p value | **After 2^nd^ dose of COVID-19 vaccine**  Frequency (%) | | | p value |
| --- | --- | --- | --- | --- | --- | --- | --- | --- |
|  | Age 18-24  N=180 | Age 25-65  N=869 | Age >66  N=37 |  | Age 18-24  N=27 | Age 25-65  N=254 | Age >66  N=18 |  |
| **Presence**  No  Yes | 110 (61.1)  70 (38.9) | 0  869 (100) | 0  37 (100.0) | < 0.001 | -  - | 35 (13.7)  248 (97.6) | 37  0 | <0.001 |
| **Injection site pain**  **Redness and swelling** | 129 (71.7)  20 (11.1) | 697 (80.2)  150 (17.3) | 25 (67.6)  7 (18.9) | 0.011  0.115 | 20 (74.0)  4 (14.8) | 169 (66.5)  37 (14.4) | 11 (61.1)  4 (22.2) | 0.006  0.054 |
| **Bone and muscle pain** | 71 (39.4) | 334 (38.4) | 3 (8.1) | 0.001 | 7 (25.9) | 73 (28.7) | 5 (27.7) | 0.052 |
| **Flu like symptoms**  Fever  Headache  Chills  Sore throat  fatigue  cough  runny nose  loss of taste  loss of smell | 50 (27.8)  55 (30.6)  55 (15.7)  15 (8.3)  89 (49.4)  23 (12.8)  12 (6.7)  2 (1.1)  2 (1.1) | 248 (28.5)  297 (34.2)  292 (33.6)  73 (8.4)  461 (53.1)  67 (7.7)  63 (7.2)  11 (1.3)  13 (1.5) | 2 (5.4)  7 (18.9)  4 (10.8)  2 (5.4)  13 (35.1)  1 (2.7)  1 (2.7)  1 (2.7)  1 (0.5) | 0.009  0.114  0.013  0.811  0.078  0.037  0.559  0.730  0.759 | 8 (29.6)  5 (18.5)  7 (25.9)  2 (7.4)  13 (48.1)  2 (7.4)  3 (11.1)  0  0 | 46 (18.1)  53 (20.8)  58 (22.8)  14 (5.5)  102 (4.1)  8 (3.1)  9 (3.5)  2 (0.7)  3 (1.1) | 3 (16.6)  1 (5.5)  4 (22.2)  2 (11.1)  6 (33.3)  0  2 (11.1)  0  0 | 0.655  0.153  0.200  0.171  0.131  0.812  0.062  0.779  0.687 |
| **GI side effect**  Nausea  Diarrhea  Vomiting  loss of appetite  abdominal pain  constipation | 29 (16.1)  11 (6.1)  2 (1.1)  20 (11.1)  15 (8.3)  3 (1.7) | 134 (15.4)  59 (6.8)  28 (3.2)  82 (9.8)  50 (5.8)  4 (0.5) | 1 (2.7)  0  0  0  0  0 | 0.098  0.252  0.168  0.111  0.122  0.162 | 2 (7.4)  2 (7.4)  2 (7.4)  4 (14.8)  1 (3.7)  0 | 19 (7.4)  10 (3.9)  6 (2.3)  13 (5.1)  6 (2.3)  2 (0.7) | 2 (11.1)  1 (5.5)  0  2 (11.1)  0  0 | 0.243  0.692  0.724  0.179  0.865  0.779 |
| **Psychological SE**  Sleep disturbance  Anxiety and stress  Depression | 22 (12.2)  17 (9.4)  12 (6.7) | 87 (10.0)  51 (5.9)  36 (4.1) | 1 (2.7)  0  0 | 0.210  0.055  0.134 | 3 (11.1)  2 (7.4)  1 (3.7) | 17 (6.6)  6 (2.3)  4 (1.5) | 2 (11.1)  0  0 | 0.322  0.724  0.902 |
| **Irregular menses** | 2 (1.1) | 14 (1.6) | 1 (2.7) | 0.754 | 1 (3.7) | 3 (1.1) | 0 | 0.852 |
| **Decreased libido** | 2 (1.1) | 12 (1.4) | 0 | 0.746 | 0 | 5 (1.9) | 0 | 0.534 |
| **Lymphnode enlargement & swelling** | 2 (1.1) | 9 (1.0) | 1 (2.7) | 0.637 | 0 | 4 (1.5) | 0 | 0.606 |
| **Skin itching**  **Acne**  **Sweating** | 5 (2.8)  3 (1.7)  12 (6.7) | 24 (2.8)  17 (2.0)  64 (7.4) | 0  2 (5.4)  0 | 0.591  0.322  0.224 | 0  0  2 (7.4) | 6 (2.3)  3(1.1)  8 (3.1) | 0  2 (11.1)  1 (5.5) | 0.471  <0.0010.564 |
| **Increase urination**  **Change urine color** | 1 (0.6)  3 91.7) | 15 (1.7)  8 (0.9) | 0  0 | 0.372  0.543 | 0  0 | 2 (0.2)  2 (0.7) | 0  0 | 0.779  0.779 |
| **Blurred vision** | 7 (3.9) | 33 (3.8) | 0 | 0.480 | 1 (3.7) | 6 (2.3) | 1(5.5) | 0.357 |
| **Cardiac side effects**  Shortness of breath  Palpitation  Chest pain  Loss of consciousness  Paleness  Bleeding anywhere | 15 (8.3)  19 (10.6)  10 (5.6)  2 (1.1)  5 (2.8)  2 (1.1) | 61 (7.0)  74 (8.5)  46 (5.3)  5 (0.6)  30 (3.5)  3 (0.3) | 0  0  0  0  0  0 | 0.194  0.112  0.349  0.632  0.472  0.352 | 2 (7.4)  1 (3.7)  2 (7.4)  0  1(3.7)  0 | 18 (7.0)  8 (3.1)  8 (3.1)  0  5 (1.9)  0 | 1 (5.5)  1 (5.5)  1 (5.5)  0  0  0 | 0.656  0.460  0.564  -  0.899  - |
| Dizziness | 26 (14.4) | 128 (14.7) | 3 (8.1) | 0.533 | 2 (7.4) | 23 (9.0) | 2 (11.1) | 0.247 |
| **Require hospitalization** | 0 | 0 | 0 |  | 0 | 2 (0.7) | 0 | 0.821 |
| **Onset of Symptoms**  Immediately  1^st^ day  1^st^ week  2^nd^ week  3^rd^ week | 20 (13.0)  116 (75.3)  12 (7.8)  5 (3.2)  1 (0.6) | 72 (9.3)  644 (82.9)  53 (6.8)  5 (0.6)  3 (0.4) | 3 (11.1)  21 (77.8)  3 (11.1)  0  0 | 0.128 | 2 (7.4)  17 (62.9)  4 (14.8)  0  0 | 24 (9.4)  162 (0.6)  16 (6.3)  35 (13.7)  2 (0.7) | 1 (5.5)  11 (61.1)  1 (5.5)  3 (16.6)  0 | 0.676 |
| **Duration of symptoms** | 3.67 (5.27) | 2.32 (3.02) | 2.25 (1.38) | 0.363 | 2.13 (1.59) | 2.76 (4.11) | 3.13 (2.29) | 0.065 |

N: population size, SD: standard deviation. *P* value < 0.05 is statistically significant

**Supplementary Table 2 -Side effect after receiving vaccines related to influenza vaccine**

| **Symptoms** | **After 1^st^ dose of COVID-19 vaccine**  Frequency (%) | | | **After 2^nd^ dose of COVID-19 vaccine**  Frequency (%) | | p value |
| --- | --- | --- | --- | --- | --- | --- |
|  | Influenza vaccine | | P value | Influenza vaccine | |  |
|  | Yes  N= 264 | No  N= 813 |  | Yes  N= 102 | No  N= 195 |  |
| **Presence**  No  Yes | 19 (7.2)  245 (92.8) | 91 (11.2)  722 (88.8) | 0.037 | 20 (19.6)  79 (77.4) | 28 (14.4)  167 (85.6) | 0.485 |
| **Injection site pain**  **Redness and swelling** | 199 (75.4)  46 (17.4) | 646 (79.5)  128 (15.7) | 0.095  0.519 | 76 (74.5)  22 (21.5) | 123 (63.0)  23 (22.4) | <0.001<0.001 |
| **Bone and muscle pain** | 92 (34.8) | 312 (38.4) | 0.170 | 33 (32.3) | 51 (26.1) | 0.001 |
| **Flu like symptoms**  Fever  Headache  Chills  Sore throat  fatigue  cough  runny nose  loss of taste  loss of smell | 63 (23.9)  82 (31.1)  77 (29.2)  26 (9.8)  120 (45.5)  23 (8.7)  5 (1.9)  3 (1.1)  5 (1.9) | 233 (28.7)  274 (33.7)  272 (33.5)  64 (7.9)  438 (53.9)  68 (8.4)  9 (1.1)  11 (1.4)  11 (1.4) | 0.074  0.237  0.111  0.188  0.010  0.473  0.243  0.538  0.352 | 20 (19.6)  24 (23.5)  27 (26.4)  7 (6.8)  44 (43.1)  1 (0.9)  5 (4.9)  0  0 | 35 (17.9)  34 (17.4)  41 (21.0)  11 (5.6)  75 (38.4)  9 (4.6)  9 (4.6)  2 (1.0)  3 (1.5) | 0.036  0.002  0.003  0.153  0.001  0.284  0.327  0.420  0.323 |
| **GI side effect**  Nausea  Diarrhea  Vomiting  loss of appetite  abdominal pain  constipation | 31 (11.7)  18 (6.8)  5 (1.9)  18 (7.1)  16 (6.1)  3 (1.1) | 132 (16.2)  52 (6.4)  25 (3.1)  84 (10.6)  49 (6.0)  4 (0.5) | 0.045  0.453  0.216  0.062  0.542  0.233 | 9 (8.8)  5 (4.9)  2 (1.9)  9 (8.8)  2 (1.9)  1 (0.9) | 14 (7.1)  8 (4.1)  6 (3.0)  10 (5.1)  5 (2.5)  1 (0.5) | 0.099  0.239  0.974  0.019  0.802  0.402 |
| **Psychological SE**  Sleep disturbance  Anxiety and stress  Depression | 22 (8.3)  12 (4.5)  9 (3.4) | 87 (10.7)  56 (6.9)  39 (4.8) | 0.161  0.110  0.222 | 8 (7.8)  4 (3.9)  1 (0.9) | 14 (13.7)  4 (2.0)  4 (2.0) | 0.192  0.093  0.814 |
| **Irregular menses** | 4 (1.5) | 13 (1.6) | 0.593 | 2 (1.9) | 2 (1.0) | 0.235 |
| **Decreased libido** | 5 (1.9) | 9 (1.1) | 0.243 | 0 | 5 (2.5) | 0.202 |
| **Lymph node enlargement & swelling** | 2 (0.8) | 10 (1.2) | 0.405 | 2 (1.9) | 2 (1.0) | 0.235 |
| **Skin itching**  **Acne**  **Sweating** | 3 (1.1)  7 (2.7)  22 (8.3) | 26 (3.2)  15 (1.8)  53 (6.5) | 0.072  0.421  0.192 | 2 (1.9)  0  4 (3.9) | 4 (2.0)  5 (2.5)  7 (3.5) | 0.614  0.202  0.358 |
| **Increase urination**  **Change urine color** | 6 (2.3)  2 (0.8) | 10 (1.2)  9 (1.1) | 0.224  0.624 | 1 (0.9)  0 | 1 (0.5)  2 (1.0) | 0.402  0.420 |
| **Blurred vision** | 9 (3.4) | 31 (3.8) | 0.763 | 2 (1.9) | 5 (2.5) | 0.802 |
| **Cardiac side effects**  Shortness of breath  Palpitation  Chest pain  Loss of consciousness  **Paleness**  **Bleeding anywhere** | 20 (7.6)  25 (9.5)  12 (4.5)  2 (0.8)  8 (3.0)  0 | 56 (6.9)  68 (8.4)  44 (5.4)  5 (0.6)  26 (3.2)  5 (0.6) | 0.398  0.329  0.356  0.543  0.892  0.202 | 11 (10.7)  3 (2.9)  4 (3.9)  0  1 (0.9)  0 | 10 (5.1)  6 (3.0)  7(3.5)  0  5 (2.5)  0 | 0.003  0.537  0.358  0.654 |
| **Dizziness** | 32 (12.1) | 124 (15.3) | 0.123 | 12 (11.7) | 14 (13.7) | 0.009 |
| **Require hospitalization** | 0 | 0 |  | 1 (0.9) | 1 (0.5) | 0.647 |
| **Onset of Symptoms**  Immediately  1^st^ day  1^st^ week  2^nd^ week  3^rd^ week | 19 (8.4)  187 (83.1)  17 (7.6)  2 (0.9)  0 | 75 (10.3)  589 (81.2)  49 (6.8)  8 (1.1)  4 (0.6) | 0.704 | 11 (10.7)  68 (66.6)  8 (7.8)  9 (8.8)  2 (1.9) | 15 (7.7)  121 (62.0)  13 (6.7)  29 (14.8)  1 (0.5) | 0.256 |
| **Duration of symptoms mean (SD)** | 1.90 (1.32) | 2.68 (3.68) | 0.744 | 1.95 (1.45) | 3.17 (4.70) | 0.295 |

N: population size, SD: standard deviation. *P* value < 0.05 is statistically significant

**Supplementary Table 3- Side effect after receiving vaccines related to health condition**

| **Symptoms** | **After 1^st^ dose of COVID-19 vaccine** Frequency (%) | | | **After 2^nd^ dose of COVID-19 vaccine**  Frequency (%) | | |
| --- | --- | --- | --- | --- | --- | --- |
|  | Health condition | | p value | Health condition | | P value |
|  | Yes  N= 230 | No  N= 856 |  | Yes  N=72 | No  N=227 |  |
| **Presence**  No  Yes | 23 (10.0)  207 (90.0) | 87 (10.2)  769 (89.8) | 0.942 | 17 (23.6)  46 (63.8) | 55 (24.2)  202 (88.9) | 0.342 |
| **Injection site pain**  **Redness and swelling** | 163 (70.9)  38 (16.5) | 688 (80.4)  139 (16.2) | 0.002  0.918 | 40 (55.6)  13 (18.0) | 160 (70.5)  32 (14.1) | 0.652  0.196 |
| **Bone and muscle pain** | 90 (39.1) | 318 (37.1) | 0.317 | 22 (30.6) | 63 (27.7) | 0.269 |
| **Flu like symptoms**  Fever  Headache  Chills  Sore throat  fatigue  cough  runny nose  loss of taste  loss of smell | 58 (25.2)  76 (33.0)  80 (34.8)  19 (8.3)  115 (50.0)  22 (9.6)  20 (8.7)  4 (1.7)  6 (2.6) | 242 (28.3)  283 (33.1)  271 (31.7)  71 (8.3)  448(52.4)  69 (8.1)  56 (6.5)  10 (1.2)  10 (1.2) | 0.358  0.532  0.206  0.556  0.284  0.271  0.161  0.342  0.101 | 12 (16.6)  17 (23.6)  16 (22.2)  8 (11.1)  30 (41.6)  2 (2.8)  4 (5.5)  1 (1.4)  1 (1.4) | 45 (19.8)  42 (18.5)  53 (23.3)  10 (4.4)  91 (40.0)  8 (3.5)  10 (4.4)  1 (0.4)  2 (0.9) | 0.981  0.140  0.673  0.015  0.302  0.927  0.496  0.318  0.606 |
| **GI side effect**  Nausea  Diarrhea  Vomiting  loss of appetite  abdominal pain  constipation | 32 (13.9)  15 (6.5)  4 (1.7)  16 (7.1)  17 (7.4)  4 (1.7) | 132 (15.4)  55 (6.4)  26 (3.0)  86 (10.3)  48 (5.6)  3 (0.4) | 0.326  0.529  0.204  0.089  0.194  0.040 | 5 (6.9)  3 (4.1)  1 (1.4)  7 (9.7)  1 (1.4)  0 | 18 (7.9)  10 (4.4)  7 (3.0)  12 (5.2)  6 (2.7)  2 (0.9) | 0.947  0.866  0.546  0.092  0.654  0.463 |
| **Psychological SE**  Sleep disturbance  Anxiety and stress  Depression | 21 (9.1)  16 (7.0)  10 (4.3) | 89 (10.4)  52 (6.1)  38 (4.4) | 0.335  0.360  0.560 | 8 (11.1)  2 (2.8)  0 | 14 (6.1)  6 (2.7)  5 (2.2) | 0.078  0.791  0.245 |
| **Irregular menses** | 2 (0.9) | 15 (1.8) | 0.267 | 0 | 4 (1.7) | 0.299 |
| **Decreased libido** | 3 (1.3) | 11 (1.3) | 0.596 | 0 | 5 (2.2) | 0.245 |
| **Lymphnode enlargement & swelling** | 3 (1.3) | 9 (1.1) | 0.484 | 1 (1.4) | 3 (1.3) | 0.851 |
| **Skin itching**  **Acne**  **Sweating** | 5 (2.2)  3 (1.3)  21 (9.1) | 24 (2.8)  19 (2.2)  55 (6.4) | 0.599  0.382  0.102 | 3 (4.1)  0  5 (6.9) | 3 (1.3)  5 (2.1)  6 (2.7) | 0.083  0.245  0.048 |
| **Increase urination**  **Change urine color** | 3 (1.3)  1 (0.4) | 13 (1.5)  10 (1.2) | 0.811  0.324 | 1 (1.4)  0 | 1 (0.4)  2 (0.8) | 0.318  0.463 |
| **Blurred vision** | 13 (5.7) | 27 (3.2) | 0.074 | 5 (6.9) | 3 (1.3) | 0.004 |
| **Cardiac side effects**  Shortness of breath  Palpitation  Chest pain  Lossof consciousness  Paleness  **Bleeding anywhere** | 21 (9.1)  15 (6.5)  13 (5.7)  1 (0.4)  12 (5.2)  1 (0.4) | 55 (6.4)  78 (9.1)  43 (5.0)  6 (0.7)  23 (2.7)  4 (0.5) | 0.102  0.131  0.404  0.544  0.054  0.948 | 9 (12.5)  6 (8.3)  5 (6.9)  0  3 (4.1)  0 | 12 (5.2)  4 (1.7)  6 (2.7)  0  3 (1.3)  0 | 0.014  0.003  0.048  0.083 |
| **Dizziness** | 40 (17.4) | 117 (13.7) | 0.095 | 9 (12.5) | 18 (7.9) | 0.117 |
| **Require hospitalization** | 0 | 0 |  | 1 (1.4) | 1 (0.4) | 0.340 |
| **Onset of Symptoms**  Immediately  1^st^ day  1^st^ week  2^nd^ week  3^rd^ week | 15 (7.8)  157 (81.3)  17 (8.8)  2 (1.0)  2 (1.0) | 80 (10.5)  624 (81.6)  51 (6.7)  8 (1.0)  2 (0.3) | 0.363 | 3 (4.1)  41 (56.9)  6 (8.3)  8 (11.1)  1(1.4) | 24 (10.5)  149 (65.6)  15 (6.6)  30 (13.2)  1 (0.4) | 0.276 |
| **Duration of symptoms mean SD)** | 3.60 (5.88) | 2.10 (1.97) | 0.058 | 3.86 (5.51) | 2.45 (3.34) | 0.017 |

N: population size, SD: standard deviation. *P* value < 0.05 is statistically significant

**Supplementary Table 4- Side effect after receiving vaccines related to allergy status**

| **Symptoms** | **After 1^st^ dose of COVID-19 vaccine**  Frequency (%) | | | | p value | **After 2^nd^ dose of COVID-19 vaccine**  Frequency (%) | | | | p value |
| --- | --- | --- | --- | --- | --- | --- | --- | --- | --- | --- |
|  | No allergy  N=805 | Seasonal  N=232 | Food  N=23 | Penicillin  N=25 |  | No allergy  N=227 | Seasonal  N= 63 | Food  N=5 | Penicillin  N=4 |  |
| **Presence**  No  Yes | 80 (9.9)  725 (90.1) | 27 (11.6)  205 (88.4) | 1 (4.3)  22 (95.7) | 2 (8.0)  23 (92.0) | 0.664 | 53 (23.3)  185 (81.4) | 17 (26.9)  53 (84.1) | 1 (20.0)  4 (80.0) | 1 (25.0)  3 (75.0) | 0.935 |
| **Injection site pain**  **Redness and swelling** | 618 (76.8)  127 (15.8) | 192 (82.8)  38 (16.4) | 21 (91.3)  6 (26.1) | 19 (76.0)  6 (24.0) | 0.102  0.413 | 153 (59.4)  32 (14.0) | 42 (66.6)  9 (14.3) | 3 (60.0)  2 (40.0) | 2 (50.0)  2 (50.0) | 0.485  0.526 |
| **Bone and muscle pain** | 280 (34.8) | 105 (45.3) | 12 (52.2) | 10 (40.0) | 0.014 | 61 (26.8) | 20 (31.7) | 3 (60.0) | 1 (25.0) | 0.649 |
| **Flu like symptoms**  Fever  Headache  Chills  Sore throat  fatigue  cough  runny nose  loss of taste  loss of smell | 208 (25.8)  239 (29.7)  242 (30.1)  64 (8.0)  397 (49.4)  58 (7.2)  52 (6.5)  12 (1.5)  11 (1.4) | 72 (31.0)  98 (42.2)  83 (35.8)  20 (8.6)  133 (57.3)  27 (11.6)  17 (7.3)  2 (0.9)  5 (2.2) | 11 (47.8)  9 (39.1)  14 (60.9)  1 (4.3)  15 (65.2)  1 (4.3)  2 (8.7)  0  0 | 8 (32.0)  13 (52.0)  11 (44.0)  5 (20.0)  17 (68.0)  5 (20.0)  5 (20.0)  0  0 | 0.056  0.001  0.004  0.162  0.029  0.023  0.072  0.749  0.677 | 44 (19.3)  8 (3.5)  55(24.2)  10 (4.4)  91 (40.0)  6 (2.6)  8 (3.5)  1 (0.4)  2 (0.8) | 10 (15.8)  3 (4.7)  12 (19.0)  8 (12.7)  26 (41.2)  4 (6.3)  5 (7.9)  1 (1.5)  1 (1.5) | 2 (40.0)  0  1 (20.0)  0  3 (60.0)  0  0  0  0 | 1 (25.0)  0  1 (25.0)  0  1 (25.0)  0  1 (25.0)  0  0 | 0.774  0.879  0.743  0.101  0.707  0.502  0.301  0.798  0.949 |
| **GI side effect**  Nausea  Diarrhea  Vomiting  loss of appetite  abdominal pain  constipation | 103 (12.8)  50 (6.2)  25 (3.1)  68 (8.7)  38 (4.7)  5 (0.6) | 51 (22.0)  17 (7.3)  5 (2.2)  27 (12.0)  22 (9.5)  1 (0.4) | 2 (8.7)  2 (8.7)  0  5 (21.7)  3 (13.0)  0 | 8 (32.0)  0  0  2 (8.0)  2 (8.0)  1 (4.0) | <0.0010.512  0.566  0.103  0.023  0.194 | 16 (7.0)  10 (4.4)  5 (2.2)  11 (4.8)  6 (2.6)  2 (0.8) | 6 (9.5)  3 (4.7)  2 (3.1)  7 (11.1)  1 (1.5)  0 | 0  0  0  0  0  0 | 1 (25.0)  0  1 (25.0)  1 (25.0)  0  0 | 0.745  0.893  0.261  0.262  0.896  0.874 |
| **Psychological SE**  Sleep disturbance  Anxiety and stress  Depression | 66 (8.2)  50 (6.2)  36 (4.5) | 33 (14.2)  16 (6.9)  11 (4.7) | 5 (21.7)  2 (8.7)  1 (4.3) | 6 (24.0)  0  0 | 0.001  0.559  0.749 | 15 (6.6)  7 (3.0)  5 (2.2) | 7 (11.1)  1 (1.5)  0 | 0  0  0 | 0  0  4 (100.0) | 0.523  0.838  0.626 |
| **Irregular menses** | 12 (1.5) | 3 (1.3) | 1 (4.3) | 1 (4.0) | 0.521 | 2 (0.8) | 1 (1.5) | 0 | 1 (25.0) | 0.024 |
| **Decreased libido** | 9 (1.1) | 4 (1.7) | 1 (4.3) | 0 | 0.467 | 3 (1.3) | 2 (3.1) | 0 | 0 | 0.760 |
| **Lymph node enlargement & swelling** | 8 (1.0) | 2 (0.9) | 1 (4.3) | 1 (4.0) | 0.227 | 3 (1.3) | 1 (1.5) | 0 | 0 | 0.977 |
| **Skin itching**  **Acne**  **Sweating** | 20 (2.5)  14 (1.7)  45 (5.6) | 5 (2.2)  5 (2.2)  23 (9.9) | 2 (8.7)  1 (4.3)  3 (13.0) | 2 (8.0)  2 (8.0)  5 (20.0) | 0.099  0.141  0.004 | 3 (1.3)  3 (1.3)  5 (2.2) | 2 (0.9)  2 (3.1)  6 (9.5) | 0  0  0 | 1 (25.0)  0  0 | 0.093  0.760  0.059 |
| **Increase urination**  **Change urine color** | 12 (1.5)  9 (1.1) | 4 (1.7)  2 (0.9) | 0  0 | 0  0 | 0.845  0.889 | 1 (0.4)  0 | 1 (1.5)  2 (0.9) | 0  0 | 0  0 | 0.798  0.061 |
| **Blurred vision** | 26 (3.2) | 10 (4.3) | 2 (8.7) | 2 (8.0) | 0.300 | 4 (1.6) | 4 (6.3) | 0 | 0 | 0.253 |
| **Cardiac side effects**  Shortness of breath  Palpitation  Chest pain  Loss of consciousness  Paleness  Bleeding anywhere | 48 (6.0)  60 (7.5)  36 (4.5)  5 (0.6)  26 (3.2)  2 (0.2) | 24 (10.3)  21 (9.1)  17 (7.3)  1 (0.4)  7 (3.0)  3 (1.3) | 1 (4.3)  5 (21.7)  1 (4.3)  0  1 (4.3)  0 | 3 (12.0)  7 (28.0)  2 (8.0)  1 (4.0)  1 (4.0)  0 | 0.089  <0.0010.328  0.194  0.982  0.211 | 16 (7.0)  8 (3.5)  8 (3.5)  0  5 (2.2)  0 | 5 (7.9)  1(1.5)  3(4.7)  0  1 (1.5)  0 | 0  0  0  0  0  0 | 0  1 (25.0)  0  0  0  0 | 0.797  0.325  0.879  0.941 |
| **Dizziness** | 108 (13.4) | 35 (15.1) | 7 (30.4) | 7 (28.0) | 0.026 | 20 (8.8) | 5 (7.9) | 1 (20.0) | 1 (25.0) | 0.880 |
| **Require hospitalization** | 0 | 0 | 0 | 0 |  | 2 (0.8) | 0 | 0 | 0 | 0.894 |
| **Onset of Symptoms**  Immediately  1^st^ day  1^st^ week  2^nd^ week  3^rd^ week | 71 (10.2)  572 (81.9)  42 (6.0)  10 (1.4)  3 (0.4) | 17 (7.9)  176 (81.5)  22 (10.2)  0  1 (0.5) | 3 (14.3)  18 (85.7)  0  0  0 | 4 (18.2)  14 (63.6)  4 (18.2)  0  0 | 0.148 | 22 (9.6)  143 (62.9)  13 (5.7)  29 (12.7)  2 (0.8) | 5 (7.9)  42 (66.6)  6 (9.5)  7 (11.1)  0 | 0  2 (40.0)  1 (20.0)  1(20.0)  0 | 0  3 (75.0)  1 (25.0)  1 (25.0)  0 | 0.973 |
| **Duration of symptoms** | 2.48 (3.65) | 2.15 (1.57) | 2.75 (0.50) | 2.00 (1.41) | 0.990 | 2.94 (4.45) | 2.27 (1.68) | 1.50 (1.29) | 1.50 (0.70) | 0.993 |

**Supplementary Table 5- Side effect after receiving vaccines related to history of COVID-19 infection**

| **Symptoms** | **After 1^st^ dose of COVID-19 vaccine**  Frequency (%) | | | | p value | **After 2^nd^ dose of COVID-19 vaccine**  Frequency (%) | | | | p value |
| --- | --- | --- | --- | --- | --- | --- | --- | --- | --- | --- |
|  | **Previous COVID 19 infections** | | | |  | **Previous COVID 19 infection** | | | |  |
|  | No  N=838 | Before 1^st^ dose  N=205 | After 1^st^ dose  N=38 | After 2^nd^ dose  N=5 |  | No  N=196 | Before 1^st^ dose  N= 82 | After 1^st^ dose  N=19 | After 2^nd^ dose  N=2 |  |
| **Presence**  No  Yes | 110 (13.1)  728 (86.9) | 0  205 (100) | 0  38 (100) | 0  5 (100) | <0.001 | 124 (63.2)  72 (36.8) | 29 (35.3)  53 (64.6) | 19(100)  0 | 2(100)  0 | <0.001 |
| **Injection site pain**  **Redness and swelling** | 668 (79.7)  129 (15.4) | 152 (74.1)  41 (20.0) | 27 (71.1)  5 (13.2) | 4 (80.0)  2 (40.0) | 0.235  0.180 | 151 (77.0)  28 (14.2) | 35(42.6)  12 (14.6) | 13 (68.4)  5 (26.3) | 1(50.0)  0 | 0.013  0.013 |
| **Bone and muscle pain** | 355 (42.4) | 47 (22.9) | 6 (15.8) | 0 | <0.001 | 62 (31.6) | 16 (19.5) | 6 (31.5) | 1(50.0) | 0.205 |
| **Flu like symptoms**  Fever  Headache  Chills  Sore throat  fatigue  cough  runny nose  loss of taste  loss of smell | 275 (32.8)  293 (35.0)  314 (37.5)  74 (8.8)  468 (55.8)  82 (9.8)  67 (8.0)  12 (1.4)  13 (1.6) | 21 (10.2)  59 (28.8)  31 (15.1)  14 (6.8)  78 (38.2)  8 (3.9)  8 (3.9)  1 (0.5)  2 (1.0) | 3 (7.9)  5 (13.2)  6 (15.8)  2 (5.3)  14 (36.8)  1 (2.6)  1 (2.6)  1 (2.6)  1 (2.6) | 1(20.0)  2 (40.0)  0  0  3 (60.0)  0  0  0  0 | <0.0010.019  <0.0010.613  <0.0010.022  0.122  0.621  0.847 | 45 (22.9)  51 (26.0)  54 (27.5)  14 (7.14)  94 (47.9)  9 (4.5)  10 (5.1)  2 (1.0)  3 (1.5) | 8 (9.7)  7 (8.5)  10 (12.2)  2 (2.4)  20 (24.3)  1(1.2)  2(2.4)  0  0 | 3 (15.7)  1 (5.2)  4 (21.0)  2 (10.5)  6 (31.5)  0  1(5.2)  0  0 | 1(50.0)  0  1(50.0)  0  1(50.0)  0  1(50.0)  0  2(100.0) | 0.321  0.363  0.329  0.296  0.653  0.792  0.002  0.898  0.828 |
| **GI side effect**  Nausea  Diarrhea  Vomiting  loss of appetite  abdominal pain  constipation | 149 (17.8)  61 (7.3)  30 (3.6)  92 (11.0)  56 (6.7)  6 (0.7) | 12 (5.9)  8 (3.9)  0  10 (5.7)  8 (3.9)  1 (0.5) | 3 (7.9)  1 (2.6)  0  0  1 (2.6)  0 | 0  0  0  0  0  0 | <0.0010.219  0.028  0.024  0.336  0.935 | 19 (9.6)  10 (5.1)  8 (4.0)  16 (8.1)  7 (3.5)  2 (1.0) | 2(2.4)  1(1.2)  0  1(1.2)  0  0 | 1(5.2)  2(10.5)  0  1(5.2)  0  0 | 1(50.0)  0  0  1(50.0)  0  2 (100.0) | 0.027  0.100  0.496  0.008  0.555  0.898 |
| **Psychological SE**  Sleep disturbance  Anxiety and stress  Depression | 101 (12.1)  58 (6.9)  43 (5.1) | 7 (3.4)  10 (4.9)  5 (2.4) | 2 (5.3)  0  0 | 0  0  0 | 0.002  0.244  0.180 | 19 (9.6)  8 (4.0)  5 (2.5) | 1(1.2)  0  0 | 1(5.2)  0  0 | 1(50.0)  0  0 | 0.012  0.496  0.682 |
| **Irregular menses** | 13 (1.6) | 3 (1.5) | 1 (2.6) | 0 | 0.945 | 3 (1.5) | 1 (1.2) | 0 | 0 | 0.971 |
| **Decreased libido** | 13 (1.6) | 1 (0.5) | 0 | 0 | 0.562 | 2 (1.0) | 3 (3.6) | 0 | 0 | 0.133 |
| **Lymph node enlargement & swelling** | 10 (1.2) | 0 | 2 (5.3) | 0 | 0.038 | 4 (2.0) | 0 | 0 | 0 | 0.756 |
| **Skin itching**  **Acne**  **Sweating** | 24 (2.9)  18 (2.1)  69 (8.2) | 5 (2.4)  2 (1.0)  7 (3.4) | 0  2 (5.3)  0 | 0  0  0 | 0.719  0.346  0.026 | 5 (2.5)  3 (1.5)  9 (4.5) | 1 (1.2)  0  1(1.2) | 0  2 (10.5)  1(5.2) | 0  0  0 | 0.963  0.000  0.651 |
| **Increase urination**  **Change urine color** | 13 (1.6)  9 (1.1) | 3 (1.5)  2 (1.0) | 0  0 | 0  0 | 0.878  0.925 | 2 (1.0)  2 (1.0) | 0  0 | 0  0 | 0  0 | 0.898  0.898 |
| **Blurred vision** | 37 (4.4) | 3 (1.5) | 0 | 0 | 0.124 | 6 (3.0) | 1 (1.2) | 1 (5.2) | 0 | 0.556 |
| **Cardiac side effects**  Shortness of breath  Palpitation  Chest pain  Loss of consciousness  **Paleness**  **Bleeding anywhere** | 70 (8.4)  83 (9.9)  50 (6.0)  6 (0.7)  35 (4.2)  5 (0.6) | 6 (2.9)  9 (4.4)  6 (2.9)  1 (0.5)  0  0 | 0  1 (2.6)  0  0  0  0 | 0  0  0  0  0  0 | 0.013  0.034  0.136  0.935  0.013  0.685 | 16 (8.1)  6 (3.0)  10 (5.1)  0  6 (3.0)  0 | 4(4.8)  3 (3.6)  0  0  0  0 | 1(5.2)  1(5.2)  1(5.2)  0  0  0 | 0  0  1(50.0)  0  0  0 | 0.978  0.510  0.332  0.618 |
| **Dizziness** | 138 (16.5) | 14 (6.8) | 5 (13.2) | 0 | 0.004 | 21 (10.7) | 4 (4.8) | 1 (5.2) | 1 (50.0) | 0.087 |
| **Require hospitalization** | 0 | 0 | 0 | 0 |  | 2 (1.0) | 0 | 0 | 0 | 0.830 |
| **Onset of Symptoms**  Immediately  1^st^ day  1^st^ week  2^nd^ week  3^rd^ week | 72 (9.6)  620 (82.6)  47 (6.3)  9 (1.2)  3 (0.4) | 19 (10.9)  136 (78.2)  17 (9.8)  1 (0.6)  1 (0.6) | 4 (13.8)  21 (72.4)  4 (13.8)  0  0 | 0  5 (100.0)  0  0  0 | 0.813 | 22 (11.2)  135 (68.8)  13 (6.6)  23 (11.7)  1 (0.5) | 4 (4.8)  42 (51.2)  6 (7.3)  11(13.4)  1 (1.2) | 1 (5.2)  12 (63.1)  2 (10.5)  4 (21.0)  0 | 0  1 (50.0)  0  0  0 | 0.896 |
| **Duration of symptoms mean (SD)** | 2.55 (3.73) | 2.00 (1.32) | 2.40 (1.35) |  | 0.649 | 2.59 (3.83) | 3.07 (4.44) | 3.10 (2.07) |  | 0.004 |
